# Method for Assigning Priority Levels in Acute Care (MAPLe-AC) predicts outcomes of acute hospital care of older persons - a cross-national validation

**DOI:** 10.1186/1472-6947-11-39

**Published:** 2011-06-07

**Authors:** Anja Noro, Jeffrey W Poss, John P Hirdes, Harriet Finne-Soveri, Gunnar Ljunggren, Jan Björnsson, Marianne Schroll, Palmi V Jonsson

**Affiliations:** 1 Ageing and Services Unit, National Institute for Health and Welfare, P.O. Box 30, FI-00271 Helsinki, Finland; 2 Department of Health Studies and Gerontology, University of Waterloo, 200 University Avenue West, Waterloo, N2L 3G1, Canada; 3 Homewood Research Institute, 150 Delhi Street, Guelph,ON N1E 6K9, Canada; 4 Centre for Medical Knowledge, Unit for Analyses and Comparisons, Stockholm County Council, Högbergsgatan 62, Stockholm (118 91), Sweden; 5 Department of Learning, Informatics, Management and Ethics, Karolinska Institutet, Berzelius Väg 3, Stockholm (17177), Sweden; 6 Department of Medicine, Diakonhjemmet Hospital, Pb23 Vinderen, Oslo (0319), Norway; 7 Geriatric Department, Bispebjerg Hospital, Copenhagen University, Bispebjerg Bakke 23, Copenhagen (2400), Denmark; 8 Faculty of Medicine, University of Iceland, Reykjavik, Iceland; 9 Geriatric Department, Landspitali University Hospital, Landakot 101, Reykjavik, Iceland

## Abstract

**Background:**

Although numerous risk factors for adverse outcomes for older persons after an acute hospital stay have been identified, a decision making tool combining all available information in a clinically meaningful way would be helpful for daily hospital practice. The purpose of this study was to evaluate the ability of the Method for Assigning Priority Levels for Acute Care (MAPLe-AC) to predict adverse outcomes in acute care for older people and to assess its usability as a decision making tool for discharge planning.

**Methods:**

Data from a prospective multicenter study in five Nordic acute care hospitals with information from admission to a one year follow-up of older acute care patients were compared with a prospective study of acute care patients from admission to discharge in eight hospitals in Canada. The interRAI Acute Care assessment instrument (v1.1) was used for data collection. Data were collected during the first 24 hours in hospital, including pre-morbid and admission information, and at day 7 or at discharge, whichever came first. Based on this information a crosswalk was developed from the original MAPLe algorithm for home care settings to acute care (MAPLe-AC). The sample included persons 75 years or older who were admitted to acute internal medical services in one hospital in each of the five Nordic countries (n = 763) or to acute hospital care either internal medical or combined medical-surgical services in eight hospitals in Ontario, Canada (n = 393). The outcome measures considered were discharge to home, discharge to institution or death. Outcomes in a 1-year follow-up in the Nordic hospitals were: living at home, living in an institution or death, and survival. Logistic regression with ROC curves and Cox regression analyses were used in the analyses.

**Results:**

Low and mild priority levels of MAPLe-AC predicted discharge home and high and very high priority levels predicted adverse outcome at discharge both in the Nordic and Canadian data sets, and one-year outcomes in the Nordic data set. The predictive accuracy (AUC's) of MAPLe-AC's was higher for discharge outcome than one year outcome, and for discharge home in Canadian hospitals but for adverse outcome in Nordic hospitals. High and very high priority levels in MAPLe-AC were also predictive of days to death adjusted for diagnoses in survival models.

**Conclusion:**

MAPLe-AC is a valid algorithm based on risk factors that predict outcomes of acute hospital care. It could be a helpful tool for early discharge planning although further testing for active use in clinical practice is still needed.

## Background

The challenge of care for older persons increases with the presence of multiple chronic conditions, and physical or cognitive decline, as indicated by systematic literature review by Campbell et al. 2004 [1]. Acute hospital care is a complex link in the care chain for these persons. The high cost of acute care causes pressures to shorten length of stay in this setting, but readmission rates are high, 11.8% at one month [2] and 35% with prior hospitalization during past 90 days [3]. A significant proportion of older people in acute care already have contact with home care services, and the admission to hospital increases risk for institutionalization. Clinicians in acute care are faced with the issues of taking care of these people's immediate health care needs as well as planning the hospital discharge at the earliest possible date, with or without support services.

People with multiple health care needs and underlying chronic conditions have been shown to have the poorest outcomes in hospital care [1-5]. The challenge of identifying the need for and prioritizing access to specialized geriatric services to address the needs of frail older persons in acute care is demanding. Comprehensive geriatric assessment (CGA) in in-patient settings has been shown to increase long-term chances of older persons to live at home [6,7]. Using standardized assessment for older persons at admission and using this information to support decision making would be beneficial for their discharge planning [8].

Delayed discharges are caused by medical and non-medical circumstances. Several studies have identified Activities of Daily Living (ADL) and cognitive impairments as contributors for unsuccessful discharges home [9]. Other risk factors that have been identified include delirium, incontinence, falls, pressure ulcers, psychiatric symptoms, instrumental activities of daily living deficits, certain diagnoses, severity of the illness, and lack of informal support. Based on a Cochrane systematic review by Shepperd et al. 2010 [10], a structured discharge plan tailored to the older person's needs may reduce hospital length of stay and lower the risk of readmission, but the impact of discharge planning on mortality, health outcomes, and costs still remains uncertain [9,10]. A comprehensive hospital discharge program might also be beneficial decreasing readmission and adverse outcomes [5,11,12].

Still, the question remains how to predict likely outcomes of hospital care and how people should be targeted for interventions aimed at diminishing the risk of adverse outcome of hospital care. A number of studies considering this issue have already been published [1,3-5,7,9,10,12-18]. However, none of the developed protocols, programs, or assessment tools available today act as a part of a fully integrated seamless information system for multiple care sectors.

Predictors of outcome in older medical clients are complex. Systematic data collection on admission is desirable. The interRAI - Acute Care (interRAI-AC) assessment system has been constructed to help clinicians with comprehensive geriatric assessment in acute care and to identify potentially remediable co-morbidity and functional deficiencies [3,13]. InterRAI-AC is one of the earlier versions of the interRAI instruments that have been constructed to provide a systematic approach comprehensive assessment that applies across different care settings [19].

An important element of discharge planning lies in the effectiveness of communication between hospital and community. The best use of electronic medical records highlights the importance of systematic and standardized assessment information. Recent research and development has lead to the introduction of the Method for Assigning Priority Levels for Home Care (MAPLe-HC), a decision-support tool for home care service allocation [20]. The MAPLe-HC algorithm is based on the interRAI-HC instrument [21,22]. Among home care clients, the MAPLe-HC algorithm predicted both nursing home placement in 90 days, caregiver stress, and a belief that the home care client would be better off elsewhere. The MAPLe-HC algorithm [20] was created in Canada and it was validated with data from six countries. It is now in widespread use in several Canadian provinces and Finland. Both interRAI-HC [21,22] and interRAI-AC [13] instruments have many common items that are used for the MAPLe-algorithm.

The purpose of this study was to develop the MAPLe-AC with a crosswalk from the MAPLe-HC algorithm and to test whether the new MAPLe-AC algorithm would predict outcome of acute hospital care. In this context, outcomes of interest were discharge home, nursing home placement or death, and at one year outcomes living at home or living in an institution or death, and survival.

## Methods

### Algorithm cross-walk

The basis for both MAPLe algorithms were items of standardized measurements: interRAI assessment instruments for home care (interRAI-HC) [21,22] and acute care (interRAI-AC v1.1) [13]. The interRAI-HC assessments are conducted when the new clients enter home care, and then each 6 months or earlier if the condition of the client changes. The interRAI-AC v1.1 instrument comprises longitudinal information pertinent to the whole acute care episode: pre-morbid status (30 days before admission), at admission (24 hours prior to admission), status 24 hours pre-discharge or the 7^th ^day whichever comes first, and the day when the assignment to "alternate level of care" is made for those who remain in hospital but do not require acute care. The last assessment point was not used in this study. The information concerning pre-morbid and admission status are collected during the first 24 hours in hospital and the second time on the day of discharge or the 7^th ^day of hospitalization, whichever came first. Sources of data are the elderly patients (through direct observation and/or interview), their relatives, patient records, or home care staff, whichever are available for the assessor.

**Figure 1:**
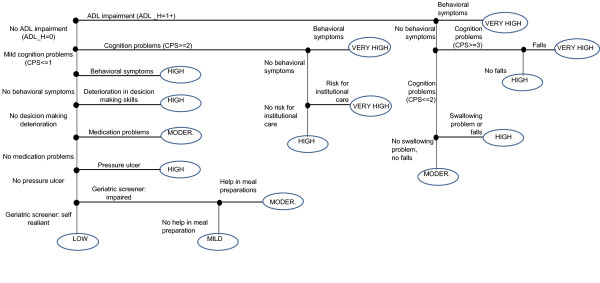
**Schematic representation of the MAPLe-AC algorithm based on client characteristics, see also Hirdes et al. 2008 **[20].

The MAPLe-AC algorithm was developed to mirror the MAPLe-HC wherever possible. The MAPLe-HC algorithm utilizes 44 interRAI-HC items [20], but only 31 of those were directly available in the acute care instrument. Figure 1 provides a schematic representation [20] of the MAPLe-AC algorithm cross-walked to the MAPLe-HC algorithm. The modification from MAPLe-HC to MAPle-AC was conducted by the original developers of MAPLe-HC and first author of this paper. The measures used in creating the algorithm were ADL impairment, cognitive impairment, behavior disturbance (verbally or physically abusive, socially inappropriate behavior, resists care), decline in decision making, problems with medication management, pressure ulcers, falls, problems with meal preparation, difficulty swallowing, and the RAI-HC's nursing home risk care-planning protocol. The items that were available in interRAI-HC but not in interRAI-AC were inadequate meals as co-classifier with swallowing problems and falls; wandering as co-classifier with risk for institutional care, environment problems as a co-classifier with medication management, and stasis ulcers as co-classifier with pressure ulcers. The Cognitive Performance Scale (CPS) [23] and ADL hierarchy [24] scales were identical. For the geriatric screener subscale, the item on stamina was missing from interRAI-AC. The risk for nursing home is calculated as a sum based on 8 categories including prior nursing home placement, going out, bladder incontinence, illnesses (Any dementia, Alzheimers' disease, Multiple Sclerosis, Head trauma), ADL decline (based on calculation in interRAI-AC), hygiene or bathing; indicators of delirium, meal and shopping activities [25]. Other items in the MAPLe-HC not available in the interRAI-AC were overall change in care needs, and difficulties in dressing. In MAPLe-AC the nursing home risk is active if there are 3 or more risks activated whereas in the MAPLe-HC the cut-off is 4 or more. The MAPLe-AC algorithm produces five main categories from low priority level to very high priority level. The MAPLe-AC algorithms were calculated for three points based on the patient's care episode information on pre-morbid, admission, and day 7 or discharge if earlier.

### Data

Two data sets were used to create and test the MAPLe-AC algorithm. The first data set was from an acute hospital care study conducted in one hospital in each of five Nordic countries: Denmark, Finland, Iceland, Norway and Sweden in 2000-2001 (later referred to as Nordic hospitals) which each served a defined geographical area [3,26]. The second data set was from a study conducted in eight acute care hospitals in the province of Ontario, Canada in 2001. The MAPLe-AC algorithm was created using the Nordic data set which subsequently was applied to the Canadian data set for validation.

In both studies the data collection was based on interRAI-AC v1.1 assessments [13]. In the Nordic study, two assessors (nurse-nurse or nurse-doctor pairs) in each country were trained by the researchers, and the interRAI-AC instrument and manual were translated from English to each of the Nordic languages by experienced translators of the interRAI tools [26]. In the Canadian study, there was one nurse assessor who was trained in doing the assessments in each hospital.

### Participants

The Nordic study was carried out in acute medical care in hospitals serving defined communities, one in each of the Nordic countries, with catchment areas of at least 90,000 persons [3,26]. The prospective study design included people aged 75 years or older (n = 763) who were admitted to hospital for acute medical care. Those in critical condition or who were admitted directly to intensive care were excluded.

Participants were randomly selected from admission lists and the patients were assessed within 24 hours with interRAI-AC instrument. The data collectors reviewed the hospital records, interviewed and observed the clients, and interviewed relatives and staff. The patients were followed up until one year after initial admission. One year follow-up data of site of residence and mortality was collected from hospital registers, contacting the person or proxy, or from national registers.

The Canadian study included people aged 75 years or older (n = 393) admitted to acute internal medical or combined medical-surgical services and assessments were done during the hospital stay and at discharge with interRAI-AC (v1.1) instrument, but without further follow-up.

### Statistical analyses

The MAPLe-AC algorithm was created for the three first assessment time points (pre-morbid, admission, and 7^th ^day or discharge), and their predictive values for various outcomes were tested in the Nordic sample. The main outcomes were either discharge home or adverse outcomes like discharge to institution or death. The one year outcomes were available only for the Nordic data and those were: living at home, or adverse outcome combining nursing home placement and/or death. The analyses involved cross-tabulations with Chi-squared tests of significance for categorical variables and means with t-tests for continuous variables (see Table 1). Tests for the predictive value of the MAPLe-AC at discharge and one year follow-up involved both bivariate and multivariate logistic regression models. The multivariate models include sex and age as dummy variables classified to four groups with the youngest age group being the reference. As a crude co-morbidity adjuster for the reason for hospital care, the following variable was used: having a new problem, exacerbation of existing problem, or both new and old problem (one or more). This variable was available in interRAI-AC (v1.1) and earlier research indicates that co-morbidities are associated with poor short- and long-term outcomes of intensive hospital care [27]. The very high priority level in MAPLe-AC was used as reference value for analyses of discharge home and living at home at one year, and the low priority level was used as reference value for adverse outcomes and in Cox regression.

**Table 1:** Basic characteristics of acute care patients and outcomes of their care between Nordic and Canadian hospitals

	**Nordic hospitals, in 2000-2001 (n = 763) **		**Canadian hospitals, in 2001 (n = 393)**		**Nordic vs. Canadian hospitals**
	**n**	**%**	**n**	**%**	**p <**
		
Women	497	65.1	234	59.5	0.267
Age 75-79	203	26.6	84	21.4	
Age 80-84	232	30.4	119	30.3	
Age 85-89	210	27.5	116	29.5	
Age 90+	118	15.5	74	18.8	0.172
					
Lived alone at admission	468	61.3	117	29.8	0.000
Earlier hospitalization (past 90 days)	239	31.3	123	31.8	0.993
					
**Reason hospitalization**					
New problem	304	39.9	211	53.7	
Exacerbation of existing problem	298	39.1	126	32.1	
Both new and old problem	160	21.0	56	14.3	0.000
					
**Outcome at discharge**					
Home	626	82.0	197	50.1	
Institution	81	10.6	109	27.7	
Dead	42	5.5	46	11.7	
Other	14	1.8	41	10.4	0.000
					
**Outcome at one year**					
Home	426	55.8	-		
Institution	79	10.4	-		
Dead	202	26.5	-		
Other	56	7.3	-		-
					
**MAPLe-AC pre-morbid**					
Low	301	39.4	124	31.6	
Mild	79	10.4	45	11.5	
Moderate	184	24.1	88	22.4	
High	151	19.8	92	23.4	
Very high	48	6.3	44	11.2	0.006
					
**MAPLe-AC admission**					
Low	91	11.9	57	14.5	
Mild	65	8.5	37	9.4	
Moderate	341	44.7	119	30.3	
High	193	25.3	118	30.0	
Very high	73	9.6	62	15.8	0.000
					
**MAPLe-AC 7^th ^day or discharge**					
Low	176	23.1	59	15.6	
Mild	101	13.7	37	9.8	
Moderate	237	31.1	105	27.8	
High	181	23.7	108	28.6	
Very high	68	8.9	69	18.3	0.000

- follow-up data not collected

ROC (Receiver Operating Character) curves have been used in visualizing a classifier's performance in order to select a suitable operating point, or decision threshold [28], and it is more commonly used in medical decision making [29]. A ROC curve is a two-dimensional depiction of classifier performance (i.e., predictive accuracy) and it indicates true positive and false positive rates. The Area Under ROC (AUC) gives the scalar value of performance varying from 0 to 1.0, and usually falls between 0.5 (50% sensitivity and 50% specificity) or 1.0 (100% sensitivity and 100% specificity). For each of the logistic analyses (SAS9.2) ROC curves were calculated of which AUC's are reported by c-statistics in Tables 2 and 3.

**Table 2:** Predicting discharge home or living at home at one year by MAPLe-AC based on pre-morbid, admission and 7^th ^day or discharge information in multivariate regression analyses adjusted for sex, age and reason for hospitalization

	Discharged home						Living home at one year		
	Nordic hospitals, in 2000-2001			Canadian hospitals, in 2001			Nordic hospitals, in 2000-2001		
n	626/137			197/196			426/295		
**Pre-morbid information**	OR^1^	95% CI^2^		OR	95% CI		OR	95% CI	

Sex (Women = 1)	1.12	0.74	1.69	1.31	0.83	2.08	1.28	0.92	1.78
Age 75-79	1.00			1.00			1.00		
Age 80-84	0.82	0.47	1.45	0.94	0.50	1.77	1.02	0.67	1.55
Age 85-89	0.86	0.49	1.52	0.50	0.26	0.94	0.92	0.59	1.41
Age 90+	0.61	0.33	1.13	0.29	0.14	3.73	0.67	0.40	1.11
*Reason for hospitalization*									
New problem	1.00			1.00			1.00		
Exacerbation of existing problem	1.46	0.91	2.32	2.26	1.37	3.73	0.79	0.55	1.13
Both new and old problem	0.76	0.47	1.25	0.71	0.35	1.43	0.77	0.50	1.19
*MAPLe-AC pre-morbid*									
Low	12.20	5.68	26.30	11.60	4.53	29.71	9.76	4.57	20.82
Mild	4.23	1.89	9.85	10.89	3.75	31.64	4.13	1.78	9.57
Moderate	2.38	1.22	4.67	5.39	2.08	13.97	3.46	1.60	7.45
High	2.08	1.05	4.12	3.10	1.20	8.03	3.44	1.57	7.55
Very high	1.00			1.00			1.00		

**Admission information**	OR	95% CI		OR	95% CI		OR	95% CI	

Sex	1.20	0.80	1.80	1.18	0.73	1.88	1.35	0.97	1.87
Age 75-79	1.00			1.00			1.00		
Age 80-84	0.74	0.43	1.28	0.88	0.46	1.67	0.91	0.60	1.38
Age 85-89	0.85	0.48	1.50	0.51	0.26	0.97	0.86	0.56	1.31
Age 90+	0.54	0.30	1.00	0.35	0.17	0.75	0.57	0.35	0.94
*Reason for hospitalization*									
New problem	1.00			1.00			1.00		
Exacerbation of existing problem	1.21	0.77	1.91	1.92	1.15	3.20	0.67	0.48	0.95
Both new and old problem	0.68	0.42	1.10	0.59	0.29	1.21	0.68	0.45	1.04
*MAPLe-AC admission*									
Low	11.63	3.77	35.88	9.39	3.84	22.94	3.51	1.74	7.10
Mild	8.77	2.84	27.13	25.80	7.60	87.64	6.14	2.76	13.69
Moderate	3.67	2.07	6.51	4.70	2.27	9.72	2.95	1.66	5.24
High	1.69	0.94	3.02	1.65	0.79	3.46	1.79	0.98	3.26
Very high	1.00			1.00			1.00		

**7^th ^day or discharge information**	OR	95% CI		OR	95% CI		OR	95% CI	

Sex	1.12	0.74	1.69	0.94	0.55	1.61	1.31	0.95	1.81
Age 75-79	1.00			1.00			1.00		
Age 80-84	0.79	0.45	1.39	0.81	0.37	1.77	0.97	0.64	1.47
Age 85-89	0.88	0.50	1.56	0.39	0.18	0.85	0.89	0.58	1.36
Age 90+	0.62	0.34	1.15	0.45	0.18	1.06	0.64	0.39	1.05
*Reason for hospitalization*									
New problem	1.00			1.00			1.00		
Exacerbation of existing problem	1.27	0.80	2.02	2.77	1.52	5.07	0.69	0.49	0.98
Both new and old problem	0.77	0.47	1.26	0.74	0.34	1.62	0.73	0.48	1.12
*MAPLe-AC 7^th ^day or discharge*									
Low	19.11	7.31	50.00	49.98	15.27	163.63	4.23	2.22	8.07
Mild	9.46	3.79	23.61	36.40	10.11	132.56	3.33	1.67	6.64
Moderate	2.83	1.59	5.06	5.83	2.57	13.23	1.77	0.98	3.20
High	2.24	1.23	4.07	4.77	2.88	10.93	1.45	0.79	2.67
Very high	1.00			1.00			1.00		

**Tests**	Pre-morbid	Admission	7^th ^day/discharge	Pre-morbid	Admission	7^th ^day/discharge	Pre-morbid	Admission	7^th ^day/discharge

R-square, %	16.5	13.1	17.0	25.0	30.6	39.2	13.9	9.0	9.7
c-statistics (AUC)	0.74	0.71	0.73	0.76	0.78	0.83	0.68	0.65	0.66

^1 ^OR = Odds Ratio, ^2 ^CI = Confidence Interval

**Table 3:** Predicting adverse outcome (death or institutionalization) at discharge and at one year by MAPLe-AC based on pre-morbid, admission and 7^th ^day or discharge information in multivariate regression analyses adjusted for sex, age and reason for hospitalization

	Adverse outcome at discharge						Adverse outcome at one year		
	Nordic hospitals, in 2000-2001			Canadian hospitals, in 2001			Nordic hospitals, in 2000-2001		
n	123/640			155/238			281/492		
**Pre-morbid information**	OR^1^	95% CI^2^		OR	95% CI		OR	95% CI	

Sex (Women = 1)	1.15	0.74	1.78	0.72	0.45	1.15	0.66	0.47	0.92
Age 75-79	1.00			1.00			1.00		
Age 80-84	1.21	0.66	2.21	0.92	0.48	1.78	1.02	0.66	1.58
Age 85-89	1.22	0.67	2.25	1.61	0.84	3.07	0.98	0.63	1.53
Age 90+	1.66	0.87	3.16	2.56	1.23	5.31	1.24	0.75	2.07
*Reason for hospitalization*									
New problem	1.00			1.00			1.00		
Exacerbation of existing problem	0.83	0.51	1.36	0.64	0.38	1.97	1.27	0.88	1.84
Both new and old problem	1.39	0.83	2.35	1.32	0.67	2.60	1.78	1.16	2.73
*MAPLe-AC pre-morbid*									
Low	1.00			1.00			1.00		
Mild	3.50	1.36	9.03	1.42	0.62	3.25	2.82	1.62	4.91
Moderate	8.58	4.13	17.83	2.71	1.42	5.16	3.45	2.24	5.31
High	9.39	4.50	19.57	5.15	2.75	9.66	5.03	3.21	7.88
Very high	19.31	8.13	45.87	10.79	4.66	25.00	17.25	7.99	37.20

**Admission information**	OR	95% CI		OR	95% CI		OR	95% CI	

Sex (women = 1)	1.06	0.69	1.64	0.81	0.51	1.31	0.64	0.46	0.88
Age 75-79	1.00			1.00			1.00		
Age 80-84	1.39	0.77	2.50	1.58	0.81	3.07	1.15	0.76	1.75
Age 85-89	1.23	0.68	2.23	2.34	1.11	4.92	1.03	0.67	1.59
Age 90+	1.93	1.02	3.65	0.79	0.47	1.34	1.43	0.87	2.36
*Reason for hospitalization*									
New problem	1.00			1.00			1.00		
Exacerbation of existing problem	1.04	0.65	1.67	0.79	0.47	1.34	1.47	1.03	2.11
Both new and old problem	1.60	0.96	2.67	1.77	0.88	3.55	1.97	1.30	2.99
*MAPLe-AC admission*									
Low	1.00			1.00			1.00		
Mild	1.97	0.32	12.20	0.42	0.10	1.68	0.89	0.39	2.02
Moderate	5.69	1.34	24.11	1.43	0.61	3.32	1.78	0.99	3.19
High	12.56	2.96	53.29	6.73	2.97	15.24	3.79	2.06	6.98
Very high	22.54	5.06	100.43	8.01	3.23	19.88	7.78	3.70	16.35

**7^th ^day or discharge information**	OR	95% CI		OR	95% CI		OR	95% CI	

Sex	1.13	0.73	1.75	0.85	0.52	1.39	0.65	0.47	0.90
Age 75-79	1.00			1.00			1.00		
Age 80-84	1.27	0.70	2.33	0.88	0.44	1.77	1.08	0.70	1.65
Age 85-89	1.17	0.64	2.14	1.36	0.68	2.69	1.02	0.66	1.57
Age 90+	1.65	0.87	3.13	2.12	0.98	4.58	1.26	0.76	2.09
*Reason for hospitalization*									
New problem	1.00			1.00			1.00		
Exacerbation of existing problem	0.99	0.61	1.61	0.78	0.46	1.35	1.44	1.00	2.06
Both new and old problem	1.41	0.84	2.37	2.00	0.99	4.06	1.82	1.20	2.77
*MAPLe-AC 7^th ^day or discharge*									
Low	1.00						1.00		
Mild	4.22	0.80	22.23				1.89	1.02	3.49
Moderate	18.06	4.29	75.99	1.00			3.30	2.01	5.43
High	25.09	5.95	105.89	4.83	2.83	8.25	5.61	3.34	9.41
Very high	51.71	11.70	228.66	11.07	5.76	21.29	8.45	4.36	16.39
									

**Tests**	Pre-morbid	Admission	7^th ^day/discharge	Pre-morbid	Admission	7^th ^day/discharge	Pre-morbid	Admission	7^th ^day/discharge

R-square, %	19.9	14.7	20.3	23.4	29.5	30.1	20.5	14.6	16.1
c-statistics (AUC)	0.76	0.73	0.76	0.75	0.78	0.78	0.73	0.69	0.70

^1 ^OR = Odds Ratio, ^2 ^Confidence Interval

**Figure 2:**
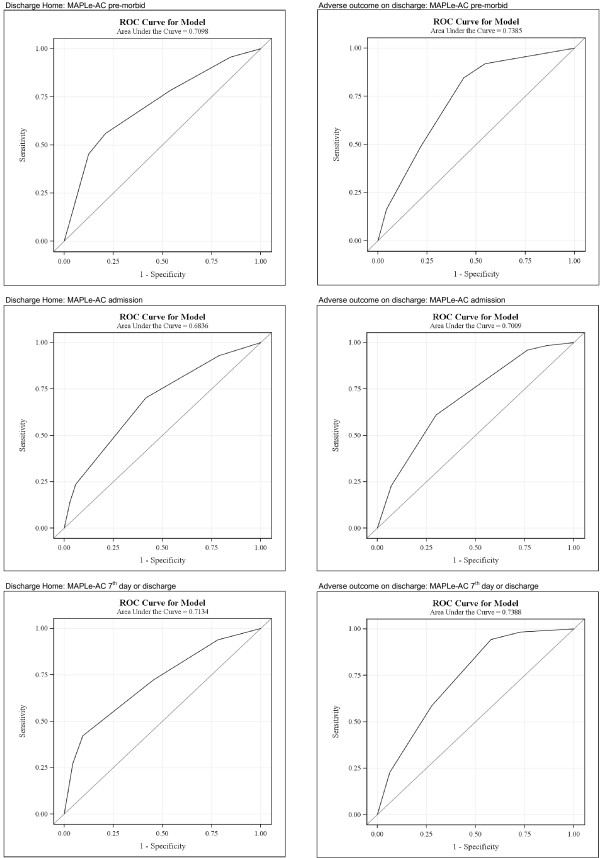
**ROC curves for discharge outcomes using MAPLe-AC algorithm as continuous variable without adjustment in logistic regression analysis**.

**Figure 3:**
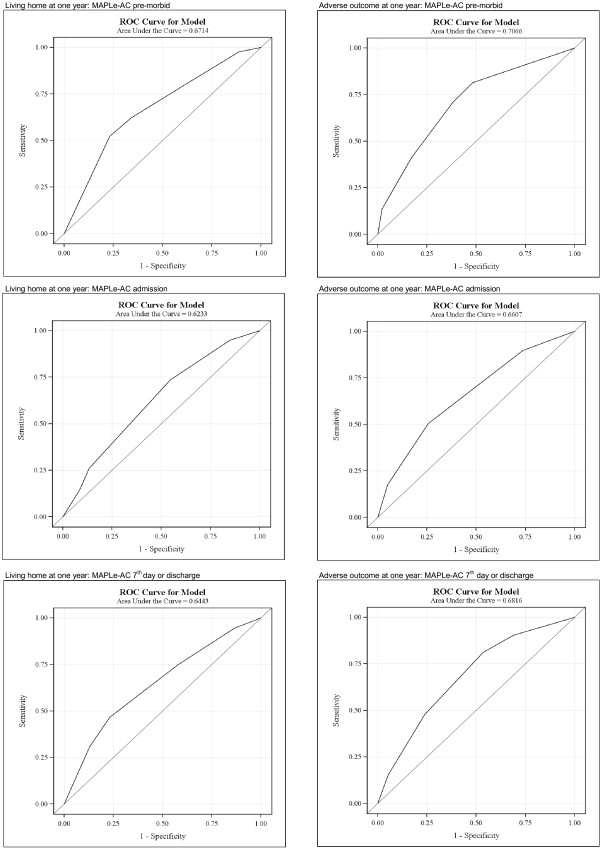
**ROC curves for one year outcome using MAPLe-AC algorithm as continuous variable without adjustment in logistic regression analysis**.

For assessing the performance of the MAPLe-AC predictions for outcomes, the ROC Curves were also calculated for MAPLe-AC's as continuous variables in bivariate models in the Nordic hospital data set. Values of AUC are presented in Table 4 and the respective ROC Curves in Figures 2 and 3.

**Table 4:** Predictive accuracy of the MAPLe-AC in predicting discharge and one year outcomes illustrated by Area under ROC curves (c-statistics).

	**Discharge outcome**		**One year outcome**	
	
	**Home**	**Adverse outcome**	**Living at home**	**Adverse outcome**
**n**	**626/137**	**123/640**	**426/295**	**281/492**
	
MAPLe-AC pre-morbid	0.71	0.74	0.67	0.71
MAPLe-AC admission	0.68	0.70	0.62	0.66
MAPLe-AC 7^th ^day or discharge	0.71	0.77	0.64	0.68

MAPLe-AC's as continuous variables without adjustment in univariate logistic regressions in Nordic sample.

Survival models for days to death (up to 365 days) were estimated with Cox regression (561 survivors and 202 deaths). Statistical analyses were conducted by using SAS for Windows 9.1 and 9.2 (SAS Institute Inc., Cary, NC, USA).

### Ethical considerations

The Nordic study plan was reviewed by ethical committees in each of the Nordic countries and informed consent was asked either from the person admitted or a proxy if the person was too sick. The pre-morbid information was collected retrospectively in the 24 hours after admission. The Canadian study plan was reviewed by University of Waterloo Office of Research Ethics, and informed consent was sought from the person admitted or proxy. The analyses conducted for this article are consistent with the purposes of the original studies, to test instrument suitability and applicability for decision making support in acute hospital care settings.

## Results

The Nordic (n = 763) and Canadian (n = 393) study populations did not statistically differ with respect to sex, age or earlier hospitalization (Table 1), or length of stay in mean + std days 12.7+21.0 vs. 14.5+18.2, p = .361. However, the Nordic patients were more likely to live alone (61%) before admission compared to the Canadians (30%). There was also significant difference with respect to the reason for admission to hospital. For half of the Canadian admissions it was a new problem (54%), and in the Nordic sample it was more often an exacerbation of an old problem (39%), or both a new and an old problem (21%).

Most of the Nordic patients were discharged home, 5.5% died during the hospital episode, and one in ten was discharged to institutional care. One in two of the Canadians were discharged home, one in three was discharged to an institution and 11.7% died during the episode. In the Nordic data set, at one year one in two persons was living at home, one in ten was living in an institution, and 27% had died (Table 1).

After creating and calculating the new MAPLe-AC algorithms for all three time points in both data sets, the priority levels were significantly higher in the Canadian sample compared with the Nordic sample. As to the pre-morbid situation, almost one in two persons was classified in a low or mild priority level, but on admission people were mostly in a moderate to high priority level, and on day 7 or at discharge there was an increase in Nordic hospitals in the proportion of low or mild priority levels compared to Canadian sample. However, a slightly higher proportion of persons were in high or very high priority levels during the pre-morbid period in the Canadian sample (Table 1).

For each time point, MAPLe-AC scores were predictive of discharge home, with similar directions and magnitudes of relationships in both data sets (Table 2). The exacerbation of an old problem was statistically significantly predictive of discharge home among persons in Canadian hospitals. In both data sets, those belonging to low or mild priority levels had the highest chance of being discharged home. There was also increased likelihood of going home for those in high priority levels compared with very high priority levels both based on pre-morbid and 7^th ^day or discharge information. When we compared Nordic and Canadian hospitals, at each assessment time the Canadian patients with moderate and high priority levels consistently had higher probability of being discharged home than the Nordic patients.

The MAPLe-AC algorithm on pre-morbid, admission, and 7^th ^day or discharge predicted living at home at one year (Table 2). The strongest predictions were found with pre-morbid information, in which those in low priority level had 9.8 times higher odds of living at home at one year. Those in high priority level had 3.4 fold higher probability of living at home than those in very high priority level. The MAPLe-AC based on admission information gave higher odds (OR = 6.14) for those in the mild priority level than those in low priority level (OR = 3.51) to live at home at 1 year compared with the very high priority level, although confidence intervals for these point estimates overlap.

The c-statistics indicating AUC's were similar with Nordic and Canadian hospitals in pre-morbid (0.74 vs. 0.76), but when using admission assessments the Canadian hospitals yielded higher values (0.78 vs. 0.71). Similar results were evident for the assessments on the 7^th ^day or discharge (0.83 vs. 0.73). For the one year outcome in Nordic hospitals, the AUC's were lower than discharge home for each measurement point (0.68 vs. 0.74; 0.65 vs. 0.71; 0.66 vs. 0.73).

MAPLe-AC predicted adverse outcomes at discharge in a similar way in both data sets, although the odds ratios were higher in the Nordic data. The likely reason is that there were fewer persons with adverse outcomes in Nordic hospitals compared with persons in Canadian hospitals (Table 3). In the pre-morbid, 7^th ^day or discharge assessments, the risk for adverse outcome was higher among those in high or very high priority levels in the pre-morbid (OR 19.31 vs. 10.79) and in 7^th ^day or discharge assessment (OR 51.7 vs. 11.1). At admission, those in moderate (OR 5.69) to very high priority levels (OR 22.5) had higher risk for adverse outcomes in Nordic hospitals, but in Canadian hospitals only those in high (OR 6.73) or very high priority levels (OR 8.01) had higher risk for adverse outcomes. The c-statistics indicating AUC's were similar with Nordic and Canadian hospitals in pre-morbid (0.76 vs. 0.75), admission (0.73 vs. 0.78) and 7^th ^day or discharge (0.76 vs. 0.78) assessments. For one year outcome in Nordic hospitals, the AUC's were a bit lower compared to the initial episode (pre-morbid 0.73 vs. 0.76; admission 0.69 vs. 0.73, 7^th ^day or discharge 0.70 vs. 0.76). When comparing AUC's regarding predicted outcome, the AUC's were higher for discharge home for Canadian hospitals and higher for adverse outcome for Nordic hospitals.

The AUC's were also estimated for MAPLe-ACs as continuous variables without adjustment. Table 4 and Figures 2 and 3 present the analyses of ROC curves illustrated by AUC (c-statistics). The AUC's for MAPLe-AC as continuous variable varied from (0.71, 0.68, 0.71) for discharge home and (0.74, 0.70, 0.77) for adverse outcome. The AUC's for one year outcomes, living at home were lower compared to discharge home AUC's (pre-morbid 0.67 vs. 0.71; admission 0.62 vs. 0.68; 7^th ^day or discharge 0.64 vs. 0.71). AUC's for adverse outcome at one year compared to adverse outcome at discharge (pre-morbid 0.71 vs. 0.74; admission 0.66 vs. 0.70; 7^th ^day or discharge (0.68 vs. 0.77) were lower. The AUCs were somewhat higher in logistic models adjusted for sex, age, and reason for hospitalization for one year outcomes (Tables 2 and 3) than when the MAPLe-AC's were used as continuous variables in the models.

The survival models indicate that MAPLe-AC is predictive of days to death (Table 5). Males had higher risk of dying sooner than women, as were those having chronic problems, or both a new and an old problem. The higher risk for death was associated with neoplasms and diseases of the respiratory system, but having a diagnosis of mental and behavioral diseases decreased likelihood of death. Having adjusted for all these conditions, MAPLe-AC in all three assessment points still had predictive power for time to death, especially for those in high or very high priority levels. In the pre-morbid assessment point those in moderate priority level also had higher hazard of dying (HR 1.76), and in the 7^th ^day or discharge assessment those in mild (HR 1.78) or moderate (HR 1.77) priority level. The hazard ratio of dying increased with each increment in priority level.

**Table 5:** Predicting death by Cox-regression models during and after hospital stay in Nordic hospitals among elderly patients (202/561) by MAPLe-AC based on pre-morbid, admission and 7^th ^day or discharge information.

	**Pre-morbid information**				**Admission information**				**7^th ^day or discharge information**			
	
	**Hazard Ratio**	**95% CI**		**p <**	**Hazard Ratio**	**95% CI**		**p <**	**Hazard Ratio**	**95% CI**		**p <**
	
Sex (Women = 1)	0.61	0.46	0.46	0.001	0.58	0.44	0.78	0.000	0.60	0.45	0.79	0.000
Age 75-79	1.00				1.00				1.00			
Age 80-84	1.17	0.79	0.79	0.428	1.23	0.83	1.83	0.299	1.18	0.80	1.75	0.409
Age 85-89	1.25	0.84	0.84	0.269	1.25	0.84	1.86	0.277	1.23	0.83	1.83	0.306
Age 90+	1.52	0.99	0.99	0.056	1.56	1.02	2.40	0.042	1.50	0.98	2.30	0.065
*Reason for hospitalization*												
New problem	1.00				1.00				1.00			
Exacerbation of existing problem	1.46	1.04	1.04	0.028	1.52	1.08	2.12	0.015	1.51	1.08	2.11	0.016
Both new and old problem	1.94	1.34	1.34	0.000	1.98	1.37	2.86	0.000	1.94	1.34	2.81	0.000
*MAPLe-AC*												
Low	1.00				1.00				1.00			
Mild	1.61	0.94	0.94	0.081	0.85	0.38	1.94	0.706	1.78	1.01	3.14	0.048
Moderate	1.76	1.19	1.19	0.005	1.31	0.76	2.25	0.328	1.77	1.11	2.82	0.017
High	2.82	1.88	1.88	0.000	2.26	1.29	3.94	0.004	2.69	1.67	4.34	0.000
Very high	3.70	2.21	2.21	0.000	2.71	1.44	5.09	0.002	3.63	2.08	6.35	0.000
*Diagnoses (ICD-10)*												
Neoplasms	3.21	2.25	2.25	0.000	3.29	2.31	4.70	0.000	3.44	2.41	4.91	0.000
Mental and behavioral diseases	0.43	0.25	0.25	0.003	0.48	0.28	0.84	0.010	0.46	0.26	0.81	0.007
Diseases of the respiratory system	1.59	1.18	1.18	0.003	1.62	1.21	2.17	0.001	1.61	1.20	2.17	0.002

## Discussion

The MAPLe-AC algorithm predicts both positive and adverse outcomes and time to death for older people, 75 years of age or older, in acute care at discharge and at one year follow-up, regardless of the country. In addition to cognitive and physical limitations, chronic conditions are of importance with regards to outcomes, especially at one year. The predictive accuracy was higher for outcomes at discharge, but lower for outcomes after one year. There were some differences between Nordic and Canadian hospitals for predicting discharge outcome. The MAPLe-AC predicted slightly better for discharge home outcome for Canadian hospitals, but for Nordic hospitals for adverse outcome at discharge.

When case-mix indexes for acute hospital care have been tested and outcomes assessed, one of the most predictive factors has been functional capacity, including both physical and cognitive function [2,15,16,18]. In calculation of the MAPLe-AC algorithm, ADLs and cognitive performance are included as they are the basic classifiers along with behavioral symptoms. In many studies, co-morbidity, falls, incontinence, nutrition, pressure ulcers, and instrumental activities of daily living (IADLs) have also been found to contribute to the explanation of use of services and outcomes of hospital care. By using these as hierarchical classifiers, MAPLe-AC takes advantage of standardized data collection and creates a hierarchical priority level decision making tree [20].

MAPLe-AC algorithm is not intended to be as a case-mix system, but rather a decision-support tool for allocation of geriatric services in acute care. The need for equivalent measures for different care providers to integrate the care of frail older people is met by this algorithm. Measures of clinical complexity like MAPLe-AC are likely to be associated with quality and costs of care over care pathways. Managing factors contributing to complexity is critical to the independence of elderly people, coordinating and funding services.

The MAPLe-AC was tested at three different time points and the question is if one of them is more suitable for use than another. Pre-morbid status reflects the status when the person was able to live at home, and it sets the baseline that possibly could be reached again after the hospital episode. Admission status has multiple variables that are in a dynamic transition between health, illness, and recovery, so the algorithm is not as stable as it is when it is based on pre-morbid status or on day 7 or discharge status. The discharge assessment is affected by many actions initiated during the hospital stay, and it occurs after a few days of improvement from the acute condition that resulted in the admission.

The Area Under ROC curves for MAPLe-AC seem to be higher for discharge status than one year outcomes. They were also higher for adverse outcomes. There may be several reasons for this. Although the MAPLe-AC algorithm does not include information on the acute illness, discharge outcome might be easier to predict due to shorter follow-up time. At one year, especially among older people, several acute episodes either for same or different reason might change the situation of the person. Additionally, chronic illnesses and/or their life conditions might change during one year. To adjust for such changes, monitoring during the ensuing year would be needed. In cases where the person enters home care services after hospital discharge this monitoring might be more easily provided. The MAPLe-AC algorithm is created based on functioning irrespective of diagnosis or treatment of the acute condition. However, in the case of older people differentiating between frailty and diagnoses is difficult [30] and thus having information on both would be helpful.

If MAPLe-AC is applied for use as a discharge planning tool in acute care hospitals for determining discharge destination it might indicate a better chance to discharge home those in low or mild priority levels at admission. The MAPLe-AC might also act as a stimulus for potential problems and also for highlighting need for rehabilitation and preventive services either during the care episode in hospital or after discharge. However, when MAPLe-AC indicates high or very high priority level, there might be an increased risk of the older person to die or end up in long-term institutional care or end up waiting for the next site of care, particularly when informal support networks are not available. Those with moderate or high priority levels might benefit from more focused support systems for them and their informal caregiver on discharge.

A combination of pre-morbid and admission status MAPLe-AC information could be considered for discharge planning - the pre-morbid status to set the priority level that might be achieved on discharge, and admission status which would reflect improvement from worsened admission status because of the acute illness. From the clinician's point of view, the earliest possible time point is desirable in predicting outcomes for triage and planning care interventions. Using pre-morbid and admission information elderly patients with higher and persistent care needs can be identified in the beginning of the acute care episode.

Decision making tools of this nature require standardized assessments and systematic data collection during each acute hospital stay. Active seeking of pre-morbid information is crucial. Well-designed integrated electronic records might be helpful. In cases where earlier admissions exist or home care services are already in use, it is easier to receive relevant information, especially if systematic and integrated assessment systems have been adopted.

There are some limitations in our study. The data were collected from different hospitals, one in each Nordic country and eight in Ontario, Canada. There may be differences in both who and how the patients are admitted to hospital and also regarding discharge what kind of support systems are available in the communities. There are country specific differences in availability of informal care, which seemed to be higher among older patients in Canadian hospitals. That might reflect to our results in which discharge home had higher predictions (AUC's where higher) for Canadian than Nordic hospitals. There might be also differences in the overall health of older population in different countries which might affect the results. However, based on the frailty and other risk factors, it seems that the MAPLe-AC algorithm can set priority levels for acute care patients, and there is certain tendency for predicting the discharge destination or death during one year.

The MAPLe-AC algorithm uses fewer items than in the original MAPLe-HC. The home environment questions, wandering, and dressing were not available in interRAI-AC instrument. The environmental questions are important when planning discharge and should be taken into consideration in addition to MAPLe-AC information, especially after a stroke or a hip fracture and among high and very high priority clients.

Further research is needed to define how best to use the information the MAPLe-AC algorithm provides for decision making in clinical practice. The MAPLe-AC algorithm should not be an automatic decision-making tool, but instead provide a chance to combine important information over different periods during the acute hospital episode, and provide information for the use of the professionals in the care pathway of the older person with acute illness.

## Conclusions

The MAPLe-AC algorithm may provide support for clinical decisions as early as at admission pointing towards interventions needed to positively modify outcomes of the hospital episode. Information from this kind of tool could also be used in discussing with the older persons the options of care and residence after hospital stay. Use of the algorithm does not compete with but supports actions that need to be taken to make the care episode and discharge successful and by so doing has a real possibility of unifying approach to similar needs and consequently enhancing quality of life of the older person.

## List of abbreviations

AC: Acute Care; ADL: Activities of Daily Living; AUC: Area Under ROC Curve; CI: Confidence Interval; CPS: Cognitive Performance Scale; HC: Home Care; HR: Hazard Ratio; IADL: Instrumental Activities of Daily Living; interRAI-AC: interRAI Acute Care Assessment Instrument; interRAI-HC: interRAI Home Care Assessment Instrument; MAPLe: Method for Assigning Priority Levels; MAPLe-HC: Method for Assigning Priority Levels - Home Care; MAPLe-AC: Method for Assigning Priority Levels - Acute Care; OR: Odds Ratio; ROC: Receiver Operating Curve

## Competing interests

The authors declare that they have no competing interests.

## Authors' contributions

AN, JWP and JPH designed, AN analyzed and wrote, JWP, JPH provided consultation with statistical methods, HFS, PVJ, GL, MS, JB critically reviewed manuscript, PJV, HFS, GL, MS, JB designed and conducted the Nordic-AC study and JWP and JPH the Canadian study. All authors read and approved the final manuscript.

## Pre-publication history

The pre-publication history for this paper can be accessed here:

http://www.biomedcentral.com/1472-6947/11/39/prepub
